# Cultural Adaptation and Validation of the Urdu Version of the Cognitive Emotion Regulation Questionnaire (CERQ) in Male Patients With Substance Use Disorders (SUDs) in Pakistan

**DOI:** 10.3389/fpsyt.2022.812075

**Published:** 2022-05-31

**Authors:** Salman Shahzad, Nasreen Bano, Nasreen Begum, Hendrée E. Jones

**Affiliations:** ^1^Institute of Clinical Psychology, University of Karachi, Karachi, Pakistan; ^2^Department of Applied Psychology, Virtual University of Pakistan, Lahore, Pakistan; ^3^Department of Obstetrics & Gynecology, University of North Carolina at Chapel Hill, Chapel Hill, NC, United States

**Keywords:** cognitive emotion regulation, self-esteem, substance use, depression, anxiety, stress

## Abstract

**Background:**

Adults with substance use disorders (SUDs) often have co-occurring mental health problems. Emotion regulation may play a vital role in mental health problems. The Cognitive Emotion Regulation Questionnaire (CERQ) is a widely used measure for assessing cognitive emotion regulation. However, it has not been used in Pakistan on patients with co-occurring SUDs and mental health issues. The present study aims to translate and adapt the CERQ into the Urdu language and to determine its reliability and convergent validity in a sample of male patients with SUDs in Pakistan.

**Method:**

Participants completed a demographic information form, the CERQ, the Depression, Anxiety, and Stress Scale Short Form [DASS-21)], and the Rosenberg Self-Esteem Scale [RSES)] in Urdu.

**Results:**

Male participants (*N* = 237) 18–50 years of age (M = 29.8, SD = 8.1) were recruited from four substance use disorder treatment centers and hospitals in Karachi. The reliability of the Urdu version of the CERQ was based on an examination of its internal consistency reliability (Cronbach's α) and test–retest reliability for both the total scale and its subscales. Internal consistency for the CERQ total (α = 0.80) was adequate, as it was for subscales of self-blame, (0.76) acceptance (0.78), rumination (0.72), positive refocusing (0.79), focus on planning (0.89), positive reappraisal (0.81), putting into perspective (0.83), catastrophizing (0.73), and other blame (0.70). The 10–14 day test–retest reliability of the CERQ total score was 0.86. Higher CERQ scores were significantly (*p*s < 0.001) negatively associated with DASS-21depression (*r* = –0.24), anxiety (*r* = –0.23), and stress (*r* = –0.27) subscales, as well as the DASS-21 total score (*r* = –0.26) and positively associated with the RSES self-esteem score (*r* = 0.30).

**Conclusion:**

The Urdu version of the CERQ is a reliable measure for investigating cognitive emotion regulation strategies related to mental health and SUDs in Pakistan.

## Introduction

In the past two decades, research on the construct of emotion regulation (ER) has grown rapidly. ER is defined as strategies to maintain, increase, or suppress a current affective state and includes the ability to regulate emotions and physiological changes to respond to a situation adequately (1). ER aims to analyze, modify, and control emotional responses, help regulate positive and negative emotions and assess any emotion's severity and extent (2, 3). Researchers have studied ER in different fields, including its application with individuals suffering from various psychopathologies [e.g., (4)].

Disruptions in the ability to regulate emotions have been linked to various psychopathologies, including anxiety and depression (5). Numerous research efforts have identified that ER may become dysfunctional when positive emotions do not balance the regulation of painful emotions. This lack of balance can lead to an inability to cope with unpleasant and persistent negative emotional states (6). These unpleasant and ongoing conditions are disturbing and may easily trigger the development of anxious and depressive symptoms (7). Individuals might try to regulate these undesired states and symptoms with psychoactive substance use and other impulsive and risky behaviors (i.e., non-suicidal self-harm injuries) (8). This emotional dysregulation may impair emotion recognition, which may further increase the risk of impulsive and psychoactive-substance-using behaviors, leading to suicidal ideation and behaviors (9). Moreover, several studies have pointed out that alexithymia (the inability to recognize or describe one's own emotions) may be a risk factor for suicide and self-harm in individuals with substance use disorders (SUDs) (10, 11).

Individuals using maladaptive cognitive emotion regulation strategies (CERs) are prone to risky behavior, including substance use disorder (12). Researchers have found that the lack of ER strategies often leads to risky behaviors and ultimately escalates negative emotions (13). For instance, Froushani and Akrami (14) found that a low ER level resulting from the inability to cope effectively and manage emotions plays a role in the onset of drug use. Shahzad et al. (15) reported that in a sample of male patients with SUDs, emotion regulation strategies predict depression.

Similarly, previous findings have indicated a strong association between mental health issues like anxiety, depression, aggression, and psychological distress with low adaptive coping strategies and the excessive utilization of maladaptive strategies (4, 16). There has been a growing interest in having mental health professionals add emotion regulation as a component of psychotherapy [e.g., (17)]. Garnefski et al. (18) have conceptualized cognitive emotion regulation (CER) as a “conscious, cognitive way of handling the intake of emotionally arousing information.” They conceptualized different CER strategies, which include; self-blame, other-blame, rumination, catastrophizing, putting into perspective, positive refocusing, positive reappraisal, acceptance, and planning (18), and have developed the cognitive emotion regulation questionnaire (CERQ) to measure these aspects of CER. Since its development, the CERQ has become a widely used measure for assessing cognitive emotion regulation.

Based on the published literature, CER may have a significant role in mental health and SUDs. It is an essential constituent in assessing patients in clinical research and practice. Researchers have asserted that clinicians must have a reliable and valid tool that can be used in different languages and cultures (19). This goal can be achieved through translating and validating a measure from the original language into other languages (20).

Since its development, the CERQ has been translated into several different languages, including Indonesian (21), Arabic (22), Spanish (23), German (24), Turkish (25), Persian (26), Chinese (27), and French (28). Subsequent research has shown that these translations have excellent psychometric properties, including good reliability and validity. However, studies have yet to examine CERQ reliability and convergent validity in a sample of patients with SUDs. The CERQ has also yet to be translated and adapted into Urdu. In line with previous studies, the present study examined the reliability and convergent validity of the CERQ administered in Urdu, in a sample of adult males with SUD in Pakistan. Construct validity of the CERQ was assessed by investigating its relationships with self-report measures of depression, anxiety and stress, and self-esteem.

## Materials and Methods

Ethical approval for the study was granted by the Departmental Ethical Review Committee, Institute of Clinical Psychology, University of Karachi, Pakistan. The concerned authorities of substance use treatment and rehabilitation centers/hospitals (i.e., Addicare Center for Treatment for Substance Use and Mental Health Problems, Alhaq Medical Center, Nai Zindagi Welfare Trust, and Parvarish Recovery Center) were approached and provided permission for the study to be conducted in their centers/hospitals. Data were collected from 01 June to 30 December 2019.

### Participants

The present study recruited only male patients with a SUD. Although reports of psychoactive substance use among females are also a growing concern in Pakistan, access to this population is challenging due to the extreme stigma and discrimination associated with substance use in females in Pakistan and the fear and shame associated with this problem among women.

Adult male patients diagnosed with SUD were recruited using a purposive sampling technique. A total of 300 participants were initially approached to participate in this study: 5 refused to participate, and 58 did not meet the criteria to participate in the study, leaving a sample of 237 participants. Inclusion criteria were a SUD diagnosis, completion of a minimum of 3 weeks in treatment, detoxification completion, clinically stable, and ability to respond. Exclusion criteria included being unable to comprehend the instructions provided to complete the research questionnaires and being unable to read and write.

### Translation Process

The authors obtained permission to translate and adapt the CERQ via email from its copyright owners.

### Expert Panel

Experts were selected according to the guidelines provided by the International Test Commission (29). These guidelines state that experts should have sufficient knowledge of both the source and target languages, both source and target cultures, the test content, and general testing principles. All four experts were bilingual with prior experience with translation, and all held a Ph.D. in clinical psychology. Two took part as experts for forward and two for backward translation.

### Forward Translation

The instrument was first independently translated into Urdu by the two forward translators. To independently translate the original English CERQ into Urdu, the translators were provided detailed information about the scale's content and the study's objectives, and the sample to be recruited. After independent translation into Urdu, the directions, items, and format of the two different Urdu versions were compared with each other and with the original English version of the scale by the expert panel. The expert panel critically evaluated the translated version to resolve any contradictions and ambiguities in the items. Items of both translations that retained Pakistani culture's conceptual, linguistic, and cultural aspects were merged into a single draft. Substitutes recommended by the experts were also taken into consideration. All the items were retained, and no items were removed in the forward translation process.

### Backward Translation

The final forward-translated version was then given to two expert translators for backward translation. These two individuals were not involved in the forward translation and were completely blind to the original version of the CERQ to minimize any bias in back-translation. These two translators independently translated the Urdu version back into English. Each item from the backward translations was analyzed and compared with the original English version of CERQ. Items after backward translation that did not retain the initial concepts were modified and rephrased by the translators. All comments given by the experts were transcribed. After a thorough evaluation of this preliminary version, a final version of the Urdu CERQ was prepared. It was then initially piloted on 30 individuals to determine feasibility. No further revisions were necessary, and the Urdu CERQ was determined to be ready to administer and test.

### Measures

Participants completed the Urdu translated version of CERQ, the Depression, Anxiety, and Stress Scale Short Form [DASS-21; (30)], and the Self-Esteem Scale [RSES; (31)]. The DASS-21 and the RSES had previously been translated into Urdu.

### Cognitive Emotion Regulation Questionnaire (CERQ)

The CERQ has 36-items, including nine conceptually distinct subscales (32). The nine subscales of the CERQ are self-blame, acceptance, rumination, positive refocusing, focus on planning, positive reappraisal, putting into perspective, catastrophizing, and other blame. Each subscale consists of 4 items, each stating what someone thinks after experiencing threatening or stressful life events. The items are measured on a 5-point Likert-type scale ranging from 1(“almost never”) to 5 (“almost always”). A CERQ total score is obtained by summing the scores on all 36 items (possible range: 36–180). Subscale scores are obtained by summing the scores of the particular subscale (possible range for each subscale: 4 to 20). Previous research in an English-speaking sample has shown that all subscales have good internal consistencies ranging from 0.68 to 0.86 (32).

### Depression Anxiety and Stress Scale, Short Form (DASS-21)

The DASS-21 is a self-report short form of the 42-item DASS (30). It is comprised of three subscales, namely, depression, anxiety, and stress. It contains 21 items measured on a 4-point scale in which “not at all” is scored as 0 and “all the time” as 3. Sample items are: “I couldn't seem to get any enjoyment out of the things I did,” “I perspired noticeably (e.g., hands sweaty) in the absence of high temperatures or physical exertion,” and “I found myself in situations that made me so anxious I was most relieved when they ended.” DASS-21 total score can range from minimum 0 to maximum 63, while scores on each subscale can range from 0 to 21. Reliability studies of the Urdu version of the DASS indicate excellent internal consistency reliability for the total score (Cronbach's α = 0.93) and respectable internal consistency reliabilities for the subscales: depression (0.84), anxiety (0.86), and stress (0.83), respectively (33).

### The Rosenberg Self Esteem Scale (RSES)

The RSES was developed by Rosenberg (31). It contains 10 self-report 4-point Likert-type items to which respondents indicate “strongly disagree” to “strongly agree.” Some items are reverse-scored so that the total score scale ranges from 0 to 30, inclusive, with higher scores indicating higher self-esteem. An Urdu version of the RSES has good internal consistency (α = 0.77) and a 4-week test re-test correlation coefficient of 0.81 (34).

### Procedure

Before enrollment in the study, potential participants were provided with an information sheet that shared the study and informed consent procedures. This sheet included the study's objective, voluntary participation, nature of confidentiality, risk, and benefits, and researcher contact details for possible contact after study completion. Data were only collected from those patients who were deemed stable. Participants completed a patient information form at the start of the study. Personal information was obtained regarding age, gender, number of siblings, education, residential area, family structure, number of family members, family income and number of earning members, drug of addiction, history, and the onset of the problem. Researchers ensured test conditions were similar for the instructions and administration of measures at their respective treatment and rehabilitation centers. Out of 237 participants, our Urdu version of the CERQ was re-administered to 47 participants, with a 10–14 days gap. The scores obtained from the same participants on the two different administrations of the test could then be compared be correlated to assess test–retest reliability.

### Statistical Analyses

Cronbach's α was used to estimate the internal consistency reliability of the CERQ and its subscales. In contrast, simple Pearson product–moment correlations were used to assess test–retest reliability, calculated for the 47 participants who were re-administered the CERQ. Convergent validity of the CERQ was determined by examining the simple Pearson product-moment correlations of the CERQ total score and subscale scores with the DASS-21 total and subscale scores and with the RSES score. All statistical analyses were conducted with the Statistical Package for Social Sciences (SPSS, V-23.0).

## Results

### Participant Characteristics

Male patients (*N* = 237) 18–50 years of age (M = 29.8, SD = 8.1) were recruited from different substance use treatment centers and hospitals in Karachi, Pakistan. Regarding marital status, 57.4% reported they were single, 40.9% married, and 1.7% divorced. Their mean years of education completed was (M = 5.3, SD = 5.5) with a monthly income in PKR (M = 24677.2, SD = 31340.5). Regarding work status, 43% were employed, 22.4% were on daily wages and worked as laborers, 14.8% had their own business, and 19.8% were unemployed. In terms of living arrangements, 44.3% lived in a nuclear family setup, and 55.7% were in a joint family setup. Regarding the type of substance use, 44% reported using heroin, 15.6% used cannabis, and 32.9% reported using multiple psychoactive substances. Regarding using psychoactive substances, 32.1% reported such use due to family-related issues (i.e., conflicts). A total of 27.8% reported started using psychoactive substances for experimentation, 27% reported use due to personal psychological issues, and 13.1% reported use due to friends using. Regarding substance use history in their family, 39.2% reported use by their immediate family members. When asked about family history of substance use, 39.2% reported that at least one immediate family member uses drugs.

Table 1 presents descriptive statistics for all self-report measures, including subscales.

**Table 1:** Descriptive statistics for the Cognitive Emotion Regulation Questionnaire (CERQ) and its subscales, the Depression Anxiety and Stress Scale, Short Form (DASS-21) and its subscales, and the Rosenberg Self-esteem Scale (RSES) (*N* = 237).

**Measures**	***Min***	***Max***	***M***	***SD***
CERQ Total	112	172	130.2	10.0
Self-blame	11	20	17.8	1.9
Acceptance	15	20	18.5	1.5
Rumination	15	20	18.4	1.5
Positive refocusing	5	20	9.5	2.2
Focus on planning	4	18	9.8	2.7
Positive reappraisal	5	18	9.4	2.3
Putting into perspective	4	16	9.3	2.0
Catastrophizing	15	20	18.5	1.5
Other blame	15	20	18.0	1.5
DASS-21 Total	3	72	43.1	14.3
Depression	0	27	14.6	5.2
Anxiety	0	25	14.0	5.3
Stress	1	23	14.4	4.6
RSES Self-esteem	10	30	15.1	4.9

*M, mean; SD, standard deviation; CERQ, Cognitive Emotion Regulation Questionnaire; DASS-21, Depression Anxiety and Stress Scale; Short Form; RSES, Rosenberg Self Esteem Scale. The CERQ total score has a possible range of 36–180, inclusive, while the CERQ subscale scores have a possible range of 4–20, inclusive. The DASS-21 total score has a possible range of 0–63, while the DASS-21 subscale scores have a possible range of 0 to 21. The RSES score has a possible range of 0–30, inclusive*.

### CERQ Reliability

Our Urdu version of CERQ had good to excellent internal consistency reliability for the total score and subscale scores (see the left-hand section of Table 2), with Cronbach's α = 0.80 for the total score and subscale reliabilities ranging from α = 0.70 (for other blame) to α = 0.89 (for focus on planning).

**Table 2:** Internal consistency reliabilities (Cronbach's α) and 10–14 day test–retest reliabilities (Pearson Product-Moment Correlations) of the CERQ total and subscale scores, and Pearson Product-Moment Correlations of the CERQ scores with the DASS-21 total and subscale scores and the Rosenberg Self-Esteem Scale (*N* = 47 for test–retest reliability; *N* = 237 otherwise).

	**Reliability**			**Convergent validity**			
				**DASS-21**			**RSES**
**Measures**	**α**	**Test-retest**	**Total Score**	**Depression**	**Anxiety**	**Stress**	
*N*	237	47	237	237	237	237	237
CERQ Total	0.80	0.86	**−0.26**	**−0.24**	**−0.23**	**−0.27**	**0.30**
Self-blame	0.76	0.85	**0.19**	**0.18**	**0.23**	0.12	**0.14**
Acceptance	0.78	0.92	−0.03	−0.04	−0.02	−0.03	−0.01
Rumination	0.72	0.95	−0.11	−0.10	−0.07	**−0.15**	0.10
Positive refocusing	0.79	0.94	**−0.33**	**−0.29**	**−0.35**	**−0.31**	**0.24**
Focus on planning	0.89	0.98	**−0.30**	**−0.26**	**−0.32**	**−0.28**	**0.19**
Positive reappraisal	0.81	0.97	**−0.23**	**−0.25**	**−0.19**	**−0.21**	**0.22**
Putting into perspective	0.83	0.99	**−0.17**	**−0.20**	**−0.14**	**−0.15**	**0.18**
Catastrophizing	0.73	0.76	−0.01	0.010	0.01	−0.04	0.15
Other blame	0.70	0.90	**0.13**	0.122	0.12	**0.13**	0.00

*CERQ, Cognitive Emotion Regulation Questionnaire; DASS-21, Depression Anxiety and Stress Scale, Short Form; RSES, Rosenberg Self Esteem Scale. All test–retest correlations are significant at p < 0.001. For the Pearson product-moment correlations (rs) of the CERQ scores with the DASS-21 total and subscale scores and RSES scores, iff |r| > 0.127, then r is significant at 0.05, while iff |r| > 0.212, then r is significant at p < 0.001. The values for those correlations whose p < 0.05 are **bolded***.

The left-hand section of Table 2 also lists the test–retest reliabilities for the CERQ total and subscale scores. Of the ten correlations, seven were equal to or >0.9, and the lowest value was 0.76 (for catastrophizing).

### CERQ Convergent Validity

Not listed in Table 2 are the internal consistency reliabilities for the DASS-21 total (Cronbach's α = 0.95) and the RSES (Cronbach α = 0.91), which were calculated to assess the reliabilities of the convergent validity measures as the indices of reliability of the two respective measures whose correlation is examined sets the lower bound for a validity coefficient. As such, if the reliabilities of the DASS-21 and the RSES were deficient, the convergent validity coefficients would have been adversely impacted, something which did not occur here.

The simple Pearson product-moment correlations of the CERQ total and subscales scores with the DASS-21 total and subscale scores and the RSES score are presented in the right-hand section of Table 2. Results for the CERQ total score indicated that it was significantly negatively associated with the DASS-21 total score and the depression, anxiety, and stress subscale scores (*p* < 0.001) and positively associated with self-esteem (*p* < 0.001). There was a clear pattern to the correlations of the CERQ subscale scores with the DASS-21 total and subscale scores and the RSES score, with the CERQ subscales that measure positive characteristics (e.g., focus of planning) showing significant negative associations with the DASS-21 total and subscale scores, and a positive association with the RSES score.

## Discussion

The aims and objectives of this study were: (1) to develop a culturally sound Urdu version of CERQ; and to investigate (2) the reliability of this Urdu version of the CERQ; and (3) its convergent validity in a sample of male patients with SUD. Convergent validity was examined regarding the relationship of the CERQ total and subscale scores with depression, anxiety, and stress and a measure of self-esteem. No normative data on these measures are available in Pakistan, but the examination of the means in relation to their respective range of scores, as well as their respective standard deviations, would suggest reasonable estimates of DASS-21 and RSES. The overall findings of this study support the strong psychometric properties of the Urdu translated version of CERQ.

The reliability and convergent validity of the Urdu version of the CERQ in a sample of adult males with SUDs would suggest that this translated measure could be used to better understand the CER strategies in this population. This better understanding of cognitive and emotional regulation may then aid in designing and implementing interventions to improve people's coping strategies with substance use disorder. Better interventions for cognitive and emotional regulation may, in turn, help improve treatment adherence, maintain a drug-free status, and overall recovery. Psycho-education of patients with SUDs, their families, and significant others about substance use could also help reduce the stigma and discrimination that creates barriers to recovery.

The present findings show an inverse relationship of CER strategies with depression, anxiety, and stress and a direct relationship with self-esteem. In the context of a substance use disorder, CER strategies, specifically maladaptive strategies, can significantly impair psychosocial functioning. So, it is vital to understand how and when these maladaptive strategies can be detrimental and intervene to overcome these problems. According to a model from Gross (35), emotion regulation plays a crucial role in health outcomes. According to Compare et al. (36), people's strategies to regulate their negative emotions are strongly associated with mental health problems such as depression; emotion dysregulation is more common in depression that impairs an individual's social skills and capacity to identify emotions and quality of life. This study's findings are consistent with previous studies, which found that inappropriate emotion regulation is an integral part of developing and maintaining depression and anxiety disorders (37, 38). Other researchers found that depression is linked with impaired cognitive control, such as difficulty accepting and processing negative material (39). There is research evidence to support this study's findings regarding the role of self-esteem in mental health outcomes (40). Results suggest that low self-esteem can lead to a lack of development and a tendency toward drugs or alcohol consumption (41). Other researchers also found the role of environmental stressors in reducing a person's well-being. Low self-esteem can contribute to various social problems like substance use, and it often plays a vital role in this regard (42).

Individuals use emotion regulation strategies to manage and reduce the consequences of the stressors (43, 44). Some research suggests that reliance on emotion-focused coping (e.g., worry, self-blame) is related to the risk of experiencing mental health issues such as anxiety, depressive symptoms, and drug use (45, 46). A study examining the influence of emotion regulation strategies in depression among male patients with SUDs found that emotion regulation strategies such as affect suppression significantly predicted depression (15). Another study of male patients treated for SUDs concluded that patients with SUDs showed a stronger tendency to use emotion-focused coping efforts, including self-criticism and problem avoidance when facing problems than patients with severe mental illness (47). Thus, patients with SUDs may be using avoidance and disengagement strategies to cope with the difficult situation in an unsuccessful way, which could negatively interfere with treatment adherence and treatment outcome. The relationship between emotion regulation strategies and SUD is necessary for the study reported here. Individuals with adaptive coping strategies respond to challenging situations to reduce the risk of substance use. Those with emotion-focused coping strategies may be less equipped to deal with stressful life situations, leading to higher chances of using drugs. Such a conclusion is consistent with Capella and associates (48) findings that male patients with SUDs who rely on avoidance coping strategies are likely to use drugs. Thus, perhaps individuals depending on emotion-focused coping strategies use drugs to deal with negative emotions and stress.

### Limitations

The present study has certain limitations related to the study design. The sample was one of convenience, and data were collected at a single time. However, the relationship of cognitive emotion regulation strategies with depression, anxiety, stress, and self-esteem could operate differently over time. Furthermore, the sample only included male patients receiving treatment for SUD. Thus, generalizability is limited to male patients without a SUD, female patients with or with a SUD, or the non-patient general population. Future research may wish to include a more diverse sample, including non-clinical samples, to understand the role of emotion regulation in improving well-being and mental health outcomes in males and females without a SUD. Future research may also attempt to disentangle the role of emotional regulation as a possible mediator between mental health issues and substance use disorder.

## Conclusions

Existing literature related to cognitive emotion regulation strategies indicates a strong link between cognitive emotion regulation with mental health problems and self-esteem. The present study showed that the Urdu version of the CERQ would be a reliable measure for patients with SUD. Using this measure may help us fill the gap in understanding the emotion regulation strategies and implementing evidence-based practices (EBPs) while linking it with the client's specific problems to improve treatment outcomes. In addition, the Urdu version of the CERQ may be helpful in the identification of adaptive and maladaptive strategies that may constitute the basis for preventive or treatment interventions specifically for patients with SUD aimed at enhancing their well-being.

## Data Availability Statement

The raw data supporting the conclusions of this article will be made available by the authors, without undue reservation.

## Ethics Statement

The study involving human participants were reviewed and approved by Departmental Ethical Review Committee, Institute of Clinical Psychology, University of Karachi, Pakistan. The patients/participants provided their written informed consent to participate in this study.

## Author Contributions

SS main researcher, design of the research, wrote part of the manuscript, and responsible for the sample selection, data collection, and data analysis. NBa wrote the introduction and discussion. NBe provided data collection, wrote part of the manuscript, and provided statistical analysis. HJ provided manuscript reviews, edits, corrections, and amendments. All authors participated and approved the study design, contributed to manuscript revision, read, and approved the submitted version.

## Conflict of Interest

The authors declare that the research was conducted in the absence of any commercial or financial relationships that could be construed as a potential conflict of interest.

## Publisher's Note

All claims expressed in this article are solely those of the authors and do not necessarily represent those of their affiliated organizations, or those of the publisher, the editors and the reviewers. Any product that may be evaluated in this article, or claim that may be made by its manufacturer, is not guaranteed or endorsed by the publisher.

## References

[1] Thompson RA. Emotion regulation: A theme in search of definition. Monograph Soc Res Child Develop. (1994) 59:25–52. 10.1111/j.1540-5834.1994.tb01276.x 7984164

[2] Gross JJ. The extended process model of emotion regulation: elaborations, applications, and future directions. Psychol Inq. (2015) 26:130–7. 10.1080/1047840X.2015.989751

[3] Koole SL. The psychology of emotion regulation: an integrative review. Cogn Emot. (2009) 23:4–41. 10.1080/02699930802619031

[4] Aldao A Nolen-Hoeksema S Schweizer S. Emotion-regulation strategies across psychopathology: a meta-analytic review. Clin Psychol Rev. (2010) 30:217–37. 10.1016/j.cpr.2009.11.004 20015584

[5] Young KS Sandman CF Craske MG. Positive and negative emotion regulation in adolescence: links to anxiety and depression. Brain Sci. (2019) 9:76. 10.3390/brainsci9040076 30934877 PMC6523365

[6] Waugh CE. The roles of positive emotion in the regulation of emotional responses to negative events. Emotion. (2020) 20:54–8. 10.1037/emo0000625 31961178

[7] Van Beveren ML Goossens L Volkaert B Grassmann C Wante L Vandeweghe L. How do I feel right now? Emotional awareness, emotion regulation, and depressive symptoms in youth. Eur Child Adoles Psychiatry. (2019) 28:389–98. 10.1007/s00787-018-1203-3 30069654

[8] Nikmanesh Z Kazemi Y Khosravy M. Study role of different dimensions of emotional self-regulation on addiction potential. J Fam Reprod Health. (2014) 8:69–72. Retrieved from: https://www.ncbi.nlm.nih.gov/pmc/articles/PMC4064769/ 24971137 PMC4064769

[9] De Berardis D Fornaro M Orsolini L Valchera A Carano A Vellante F. Alexithymia and suicide risk in psychiatric disorders: a mini-review. Front Psychiatry. (2017) 8:148. 10.3389/fpsyt.2017.00148 28855878 PMC5557776

[10] Evren C Evren B Dalbudak E Ozcelik B Oncu F. Childhood abuse and neglect as a risk factor for alexithymia in adult male substance dependent inpatients. J Psychoactive Drugs. (2009) 41:85–92. 10.1080/02791072.2009.10400677 19455912

[11] Sakuraba S Kubo M Komoda T Yamana J. Suicidal ideation and alexithymia in patients with alcoholism: a pilot study. Subst Use Misuse. (2005) 40:823–30. 10.1081/JA-200030702 15974142

[12] Vala M. Comparative study of cognitive regulation between addicted and non-addicted peoples. Eur J Forensic Sci. (2016) 3:7–10. 10.5455/ejfs.210576

[13] Weiss NH Sullivan TP Matthew TT. Explicating the role of emotion dysregulation in risky behaviors: a review and synthesis of the literature with directions for future research and clinical practice. Curr Opin Psychol. (2015) 3:22–9. 10.1016/j.copsyc.2015.01.013 25705711 PMC4332392

[14] Parker JDA Taylor R Eastabrook J Schell S Wood LM. Problem gambling in adolescence: Relationships with internet misuse, gaming abuse and emotional intelligence. Pers Individ Differ. (2008) 45:174–80. 10.1016/J.PAID.2008.03.018

[15] Shahzad S Fatima K Begum N Ali M. Emotion regulation strategies as predictors of depression in patients with substance use disorder. Pakistan J Clin Psychol. (2020) 19:17–32. Retrieved from: http://www.pjcpku.com/index.php/pjcp/article/view/3 28454522

[16] Garnefski N Kraaij V. The cognitive emotion regulation questionnaire: psychometric features and prospective relationships with depression and anxiety in adults. Eur J Psychol Assess. (2007) 23:141–9. 10.1027/1015-5759.23.3.141

[17] Berking M Ebert D Cuijpers P Hofmann SG. Emotion regulation skills training enhances the efficacy of inpatient cognitive behavioral therapy for major depressive disorder: A randomized controlled trial. Psychother Psychosom. (2013) 82:234–45. 10.1159/000348448 23712210

[18] Garnefski N Kraaij V Spinnhoven P. Negative life events, cognitive emotion regulation and emotional problems. Pers Individ Dif. (2001) 30:1311–27. 10.1016/S0191-8869(00)00113-616791542

[19] Van Windenfelt BM Treffers PDA de Beurs E Siebelink BM Koudijs E. Translation and cross-cultural adaptation of assessment instruments used in psychological research with children and families. Clin Child Fam Psychol Rev. (2005) 8:135–47. 10.1007/s10567-005-4752-1 15981582

[20] Guillemin F Bombardier C Beaton D. Cross cultural adaptation of health related quality of life measures: Literature review and proposed guidelines. J Clin Epidemiol. (1993) 46:1417–32. 10.1016/0895-4356(93)90142-N 8263569

[21] Prastuti E Wagey Tairas MM Hartini N. Adaptation and validation of cognitive emotion regulation questionnaire (CERQ) in Indonesian version. J Education Health Comm Psychol. (2020) 9:132–47.

[22] Megreya AM Latzman RD Al-Attiyah AA Alrashidi M. The robustness of the nine-factor structure of the cognitive emotion regulation questionnaire across four arabic-speaking middle eastern countries. J Cross Cult Psychol. (2016) 47:875–90. 10.1177/0022022116644785

[23] Domínguez-Sánchez FJ Lasa-Aristu A Amor PJ Holgado-Tello FP. Psychometric properties of the Spanish version of the Cognitive Emotion Regulation Questionnaire. Assessment. (2011) 20:253–61. 10.1177/1073191110397274 21467092

[24] Joormann J Stanton CH. Examining emotion regulation in depression: A review and future directions. Behav Res Ther. (2016) 86:35–49. 10.1016/j.brat.2016.07.007 27492851

[25] Tuna E Bozo Ö. The cognitive emotion regulation questionnaire: factor structure and psychometric properties of the Turkish version. J Psychopathol Behav Assess. (2012) 34:564–70. 10.1007/s10862-012-9303-8

[26] Abdi S Taban S Ghaemian A. Cognitive emotion regulation questionnaire: validity and reliability of the Persian translation of the CERQ (36-item). Proc Soc Behav. (2012) 32:2–7. 10.1016/j.sbspro.2012.01.001

[27] Zhu X Auerbach RP Yao S Abela JR Xiao J Tong X. Psychometric properties of the cognitive emotion regulation questionnaire: chinese version. Cogn Emot. (2008) 22:288–307. 10.1080/0269993070136903526925586

[28] Jermann F Van der Linden M d'Acremont M Zermatten A. Cognitive emotion regulation questionnaire (CERQ): confirmatory factor analysis and psychometric properties of the french translation. Eur J Psychol Assess. (2006) 22:126–31. 10.1027/1015-5759.22.2.126

[29] International Test Commission. (2017). The ITC Guidelines for Translating and Adapting Tests (Second edition). [www.InTestCom.org]. Available online at: https://www.intestcom.org/files/guideline_test_adaptation_2ed.pdf

[30] Lovibond SH Lovibond PF. Manual for the Depression Anxiety and Stress Scales. 2nd ed. Sydney, NSW: Psychology Foundation (1995).

[31] Rosenberg M. Society and the Adolescent Self-Image. Princeton, NJ: Princeton University Press (1965).

[32] Garnefski N Kraaij V Spinhoven P. Manual for the Use of the Cognitive Emotion Regulation Questionnaire. Karachi: Leiden University (2002).

[33] Kamal A Aslam N. Translation, Validation, and Effectiveness of Depression, Anxiety and Stress (DASS-21) in assessing the psychological distress among flood affected individuals. J Pakistan Psychiatr Soc. (2017) 14:4–20. Available online at: http://www.jpps.com.pk/article/15198028712310-Translation%20Validation%20and%20Effectiveness%20of%20Depression,%20Anxiety%20and%20stress%20scale%20(DASS-21)%20in%20Assessing%20the%20psychological%20Distress%20Among%20Flood%20Affected%20Individuals.pdf

[34] Rizwan M Malik S Malik JN Siddiqui RS. Urdu Rosenberg self–esteem scale: an analysis of reliability and validity in Pakistan. Sociol Int J. (2017) 1:56–61. 10.15406/sij.2017.01.00010

[35] Gross JJ. The emerging field of emotion regulation: an integrative review. Rev Gen Psychol. (1998) 2:271–99. 10.1037/1089-2680.2.3.271

[36] Compare A Zarbo C Shonin E Gordon WE Marconi C. Emotional regulation and depression: A potential mediator between heart and mind. Cardiovas Psychiatry Neurol. (2014) 6:324374. 10.1155/2014/324374 25050177 PMC4090567

[37] Barlow DH Allen LB Choate ML. Toward a unified treatment for emotional disorders. Behav Ther. (2004) 35:205–30. 10.1016/S0005-7894(04)80036-4 27993336

[38] Mennin DS. Emotion regulation therapy: an integrative approach to treatment-resistant anxiety disorders. J Contemp Psychother. (2006) 36:95–105. 10.1007/s10879-006-9012-2

[39] Joormann J Gotlib IH. Emotion regulation in depression: relation to cognitive inhibition. Cogn Emot. (2010) 24:281–98. 10.1080/02699930903407948 20300538 PMC2839199

[40] Ali M Shahzad S. Risk and protective factors for mental health problems in patients with substance use disorder. Pak J Psychol. (2020) 50:55–69. Retrieved from: http://www.pjpku.com/index.php/pjp/article/view/24 25054860

[41] MacArthur JD MacArthur CT. Self-Esteem. San Francisco, CA: Research Network on Socioeconomic Status and Health. (2004). In Alavi HR. The Role of Self-esteem in Tendency towards Drugs, Theft and Prostitution. Addict Health. (2011) 3:119–24. Retrieved from https://www.ncbi.nlm.nih.gov/pmc/articles/PMC3905528/24494126 PMC3905528

[42] Kahn AP Fawcett J. The Encyclopedia of Mental Health (Facts on File Library of Health and Living). 3rd ed. New York, NY: Facts on File (2007).

[43] Skinner EA Edge K Altman J Sherwood H. Searching for the structure of coping: a review and critique of category systems for classifying ways of coping. Psychol Bull. (2003) 129:216–69. 10.1037/0033-2909.129.2.216 12696840

[44] Carver CS Connor-Smith J. Personality and coping. Annu Rev Psychol. (2010) 61:679–704. 10.1146/annurev.psych.093008.100352 19572784

[45] Dashora P Erdem G Slesnick N. Better to bend than to break: coping strategies utilized by substance-abusing homeless youth. J Health Psychol. (2011) 16:158–68. 10.1177/1359105310378385 20929945

[46] Scott RM Hides L Allen JS Lubman DI. Coping style and ecstasy use motives as predictors of current mood symptoms in ecstasy users. Addict Behav. (2013) 38:2465–72. 10.1016/j.addbeh.2013.05.005 23770644

[47] Marquez-Arrico JE Río-Martínez L José Francisco Navarro JF Prat G Forero DA Adan A. Coping strategies in male patients under treatment for substance use disorders and/or severe mental illness: influence in clinical course atone-year follow-up. J Clin Med. (2019) 8:1972. 10.3390/jcm8111972 31739487 PMC6912473

[48] Capella MM Benaiges I Adan A. Neuropsychological performance in polyconsumer men under treatment. Influence of age of onset of substance use. Sci Rep. (2015) 5:12038. 10.1038/srep12038 26155725 PMC4496775

